# Artificial Intelligence-Based Software as a Medical Device (AI-SaMD): A Systematic Review

**DOI:** 10.3390/healthcare13070817

**Published:** 2025-04-03

**Authors:** Shouki A. Ebad, Asma Alhashmi, Marwa Amara, Achraf Ben Miled, Muhammad Saqib

**Affiliations:** 1 Center for Scientific Research and Entrepreneurship, Northern Border University, Arar 73213, Saudi Arabia; 2 Department of Computer Science, Faculty of Science, Northern Border University, Arar 73213, Saudi Arabiamarwa.amara@nbu.edu.sa (M.A.);; 3 Applied College, Northern Border University, Arar 73213, Saudi Arabia

**Keywords:** AI, SaMD, AI-SaMD, regulatory, systematic review

## Abstract

**Background/Objectives:** Artificial intelligence-based software as a medical device (AI-SaMD) refers to AI-powered software used for medical purposes without being embedded in physical devices. Despite increasing approvals over the past decade, research in this domain—spanning technology, healthcare, and national security—remains limited. This research aims to bridge the existing research gap in AI-SaMD by systematically reviewing the literature from the past decade, with the aim of classifying key findings, identifying critical challenges, and synthesizing insights related to technological, clinical, and regulatory aspects of AI-SaMD. **Methods**: A systematic literature review based on the PRISMA framework was performed to select the relevant AI-SaMD studies published between 2015 and 2024 in order to uncover key themes such as publication venues, geographical trends, key challenges, and research gaps. **Results**: Most studies focus on specialized clinical settings like radiology and ophthalmology rather than general clinical practice. Key challenges to implement AI-SaMD include regulatory issues (e.g., regulatory frameworks), AI malpractice (e.g., explainability and expert oversight), and data governance (e.g., privacy and data sharing). Existing research emphasizes the importance of (1) addressing the regulatory problems through the specific duties of regulatory authorities, (2) interdisciplinary collaboration, (3) clinician training, (4) the seamless integration of AI-SaMD with healthcare software systems (e.g., electronic health records), and (5) the rigorous validation of AI-SaMD models to ensure effective implementation. **Conclusions**: This study offers valuable insights for diverse stakeholders, emphasizing the need to move beyond theoretical analyses and prioritize practical, experimental research to advance the real-world application of AI-SaMDs. This study concludes by outlining future research directions and emphasizing the limitations of the predominantly theoretical approaches currently available.

## 1. Introduction

**Figure 1:**
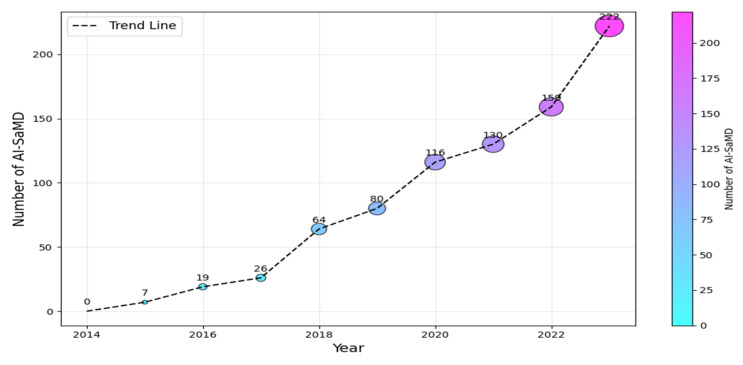
Number of AI-SaMD versus years (2015–2023).

According to the International Medical Device Regulators Forum (IMDRF (https://www.imdrf.org/)), software as a medical device (SaMD) is software intended for medical purposes independent of physical devices [1]. It operates independently of any hardware and can run on general-purpose platforms like smartphones or computers. Similarly, the US Food and Drug Administration (**FDA** (https://www.fda.gov/)**)** adopts a similar interpretation but emphasizes that SaMD must adhere to its classification rules based on risk to patients (Class I, II, or III). The importance of SaMD is evident in its growing adoption and transformative impact on healthcare. As of 2023, the global SaMD market, valued at USD 1.1 billion, was projected to experience a compound annual growth rate of over 16%, reaching USD 5.4 billion by 2032 [2,3]. In contrast, when SaMD incorporates artificial intelligence (AI) algorithms for medical purposes, it is called AI-based SaMD (hereafter, AI-SaMD). These can include diagnosing diseases, predicting patient outcomes, recommending treatments, or even detecting anomalies in medical data (such as radiographs or ECG signals). Using AI tools brings different benefits, for example, analyzing radiology images (X-rays and CT scans) to identify tumors or other abnormalities and assessing a patient’s risk of heart disease by prediction models based on historical records and genetic information [3,4]. In addition, AI algorithms automatically adjust insulin doses for diabetic patients based on real-time glucose readings. Figure 1 shows the increasing number of approved AI-SaMDs between 2015 and 2023. From the figure, it can be seen that more than 50% (472 out of 942) of AI-SaMDs were approved in the last three years, and the number of approved devices is clearly increasing every year [5]. Table 1 summarizes the key differences between SaMD and AI-SaMD.

In general, this field is still emerging, and it faces obstacles in implementation and deployment in real life. These complexities span all aspects of human health research and stem not only from technological advancements but also from other sources such as healthcare providers and governmental agencies. Research in this domain spans various fields, including social care, welfare, bioengineering, AI, machine learning, software development, and citizen science [6]. However, the literature survey revealed that there are critical areas of AI-SaMD that have not been given much attention by researchers. The focus of this paper is on these areas, aligning with the concerns and innovations driving research in this field. We provide deep insights into the theory and practice of AI-SaMD in international contexts. The paper is structured as follows. In Section 2, we briefly describe the architecture of AI-SaMD. Section 3 explains the research methodology. In Section 4, we present the results obtained and a discussion of them. Limitations of the study and future work are presented in Section 5. Finally, we conclude the paper in Section 6.

## 2. AI-SaMD Architecture

To meet quality standards, AI-SaMD’s architecture follows a multi-layered structure.

This structure, from a software-intensive systems perspective, facilitates incremental development. As layers are developed, their services can be gradually made available to users. This architecture also exhibits high changeability and portability [7]. Fundamentally, this approach organizes the system into hierarchical layers, each encapsulating related functionality. Layers provide services to those above them, with lower-level layers offering core services utilized throughout the system [7].

**Figure 2:**
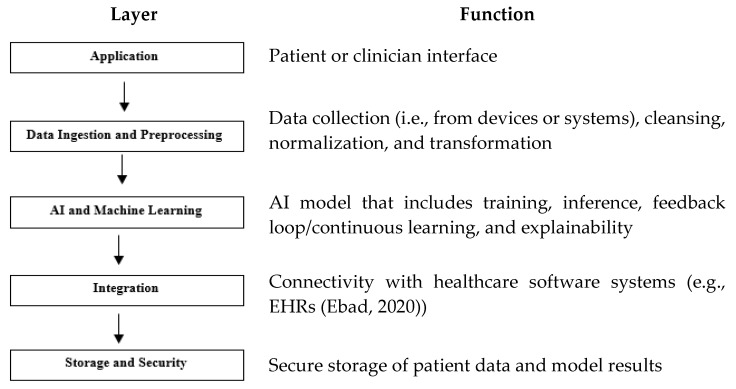
AI-SaMD layers and their functions. Note, one example [8] has shown in the function of the layer of Integration.

Figure 2 describes its layers with their functionalities. While AI-SaMD shares some architectural principles with traditional SaMD, it introduces additional layers for model training, inference, continuous learning, and explainability. These layers support the advanced capabilities of AI, making the architecture more complex but powerful in handling dynamic, data-intensive tasks.

## 3. Research Approach

A systematic literature review (SLR) is a well-defined way to evaluate, identify, synthesize, and interpret high-quality studies regarding a particular research question or a specific subject [9]. Therefore, it helps identify research gaps and suggests future research directions. This SLR was conducted based on the Preferred Reporting Items for Systematic Reviews and Meta-Analyses (PRISMA) checklist [10]. It progressed through identification, screening, eligibility, and inclusion. The identification phase retrieved relevant literature from e-libraries on 1 November 2024. During screening, titles, abstracts, and keywords were reviewed to filter initial results. The eligibility phase then assessed the full texts of potentially relevant articles. Ultimately, publications meeting the inclusion criteria were selected to characterize the AI-SaMD elements. This SLR study was performed by a team of five academic faculty members. One author developed the study protocol, which was then critically reviewed by all other team members. All researchers participated in the phases of the study.

### 3.1. Question Formulation

To refine the focus of our research, this study explores five key research questions (RQs), outlined in Table 2 along with their corresponding motivations.

### 3.2. Search Strategy

The keywords utilized to address the RQs were AI, artificial intelligence, SaMD, and software as medical device. By combining these keywords with the logical operators “AND” and “OR”, the resulting search command was constructed as follows:

(AI OR artificial intelligence) AND (SaMD OR software as medical device).

### 3.3. Source Selection

The above search command was applied to each of the search engines of the sources. By using these strings, we were able to find studies about AI-SaMD. Prior to executing the review protocol, we conducted exploratory database searches to identify optimal repositories. The selected repositories returned high-quality publications on technology and/or health from reputable, internationally recognized journals. The following five sources were used to carry out our SLR:

- PubMed;
- Semantic Scholar;
- SpringerLink;
- Web of Science (WoS);
- IEEE Explore.

### 3.4. Study Selection

Our SLR followed an iterative and incremental process. It is iterative because each search source was analyzed sequentially, completing one source before moving to the next. It is incremental in that the SLR document evolved with each iteration, expanding from an initial subset of sources to a comprehensive review. For inclusion, studies were selected based on the analysis of titles, abstracts, and keywords from the articles retrieved. Only studies written in English and published between 2015 and November 2024 were included, as this period reflects significant advancements in AI [4]. The following exclusion criteria were applied to the selected abstracts and titles:

- Exclude papers unrelated to AI-SaMD, as it is the primary focus.
- Exclude MS/PhD theses, posters, technical reports, and commentary articles.
- Exclude duplicate studies.
- Exclude non-peer-reviewed studies, such as those from preprint repositories.

**Figure 3:**
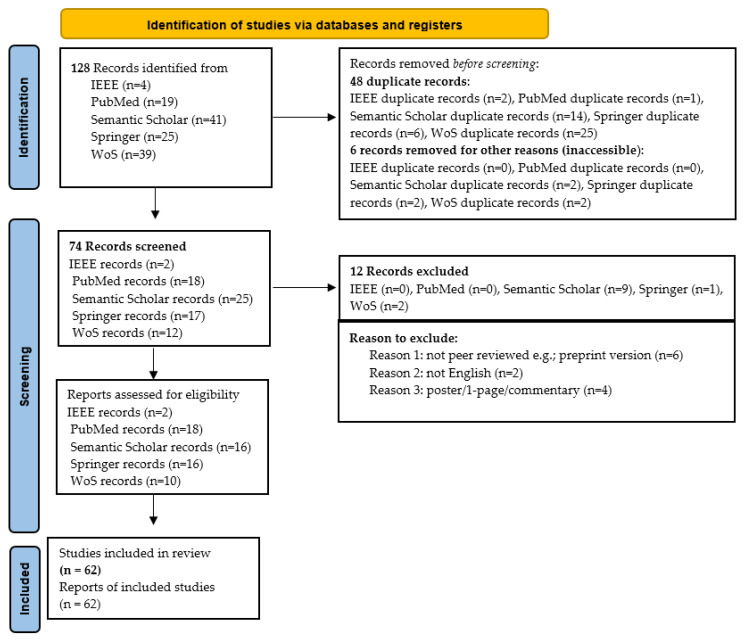
Procedure for conducting the SLR based PRISMA statement.

When ambiguity remained after reviewing the title and abstract, the full paper was examined. Figure 3 illustrates the details of how this SLR was carried out, highlighting the number of articles included and excluded at each stage. A total of 128 studies were initially identified; after applying inclusion and exclusion criteria, 62 were classified as primary studies. Table 3 provides a comprehensive breakdown of each iteration, cross-referenced with study numbers. Note that references cited within primary studies were not included in this SLR.

### 3.5. Information Extraction

As depicted in Figure 3, data extraction from the primary studies was conducted using a standardized information extraction form (Appendix A). This form, based on the search string and identified AI-SaMD studies, captured key details from each publication. The data extraction format included the following columns: study title, journal/magazine name, publication date, author(s), methodology used, study results, study objectives, country where the study was conducted, challenges associated with AI-SaMD, clinical applications or disease types where AI-SaMD was applied, data analysis details, key factors for future work, and recommendations. This structured approach ensured comprehensive information capture, facilitating an in-depth review of AI-SaMD research.

## 4. Results and Discussion

**Figure 4:**
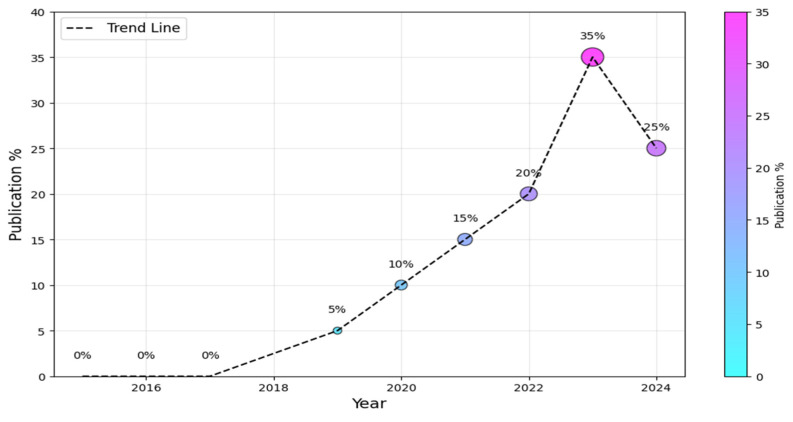
Publication trends of AI-SaMD.

To begin, we must ascertain the prevailing research trends within the AI-SaMD domain, encompassing both the information technology and healthcare sectors. Figure 4 visually depicts the annual distribution of studies. It is worth noting that the field of AI-SaMD is still emerging; that is why there was a substantial increase in publications in the second half of the period (i.e., 2019–2024) compared with the first half, where there were no publications at all. The dotted trend line suggests a positive overall trend in the percentage of publications over time, despite the slight drop in 2024. The downward trend in 2024 comes from the fact that not all empirical studies conducted in 2024 were published during performing this study, though it is still higher than earlier years. Without a doubt, this trend indicates the growing importance of AI-SaMD in recent years.

### 4.1. Analysis of Publication Venues and Source Types (RQ1)

To address RQ1, this study investigated the publication venues and source types within the field of AI-SaMD. We limited our analysis to studies published in five major libraries, listed in Table 3. These studies were found in three main publication types: conference proceedings, journal articles, and book chapters. Table 4 presents the distribution of selected studies by publication type. The percentage of studies published in journals was (c. 57, 92%), while those published in conferences, workshops, and book chapters were (c. 3, 4.8%), (c. 1, 1.6%), and (c. 1, 1.6%), respectively. It is worth mentioning that most of studies were published in journals; the rationale of this finding comes from the nature of research on AI-SaMD, which often demands a higher degree of rigor and validation, plus a relatively large space for writing. This makes journals more appropriate venues for this type of research. However, as the field grows, it is possible that one will see more specialized conferences, workshops, and symposia dedicated to the intersection of AI and healthcare, including AI-SaMD. The results of Table 4 show that (c. 50, 81%) of primary papers were retrieved from PubMed, Semantic Scholar, and Springer libraries. The contribution of the IEEE library is the smallest among all of the sources because the overall scope of this library is technological innovation but not pure healthcare like PubMed.

The results of Table 4 show that PubMed is the primary venue for publications in the AI-SaMD area, followed by Semantic Scholar and Springer. Table 5 identifies the key journals that publish papers on AI-SaMD. It displays the most common venues for the primary studies with a frequency of two or more. The results show that the *Journal of Korean Radiology* has published the highest number of studies in the current domain, with a frequency of three. This is followed by npj Digital Medicine, International Journal of Computer Assisted Radiology and Surgery, Healthcare—MDPI, Australian Journal of Dermatology, and Emergency Radiology, each with a share of two papers.

### 4.2. Demographic Trends (RQ2)

**Figure 5:**
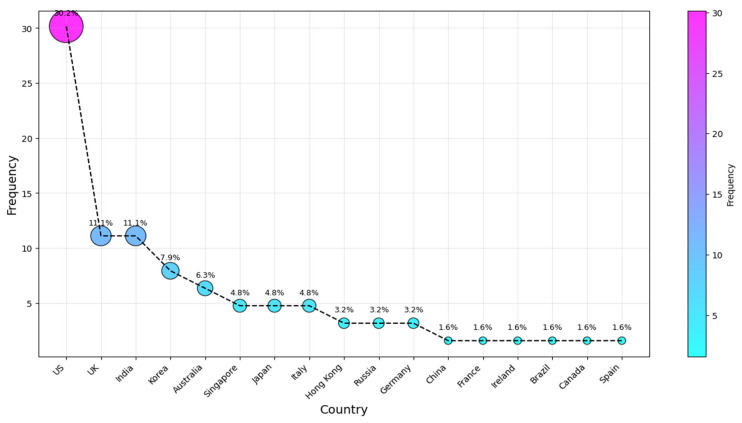
Country of publication.

To identify and rank the most active countries in AI-SaMD research, the authors’ affiliations were analyzed. This ranking aims to address RQ2 and determine the countries where researchers publishing in this field are based. We analyzed the affiliation information provided in each paper, using the country of the first author, even if the author had moved since the publication. Several review articles (e.g., [9,73]) have used this method to assess national research activity, making this a well-established bibliometric approach. Typically, the first author is the primary contributor, and their affiliation at publication reflects where the research was conducted. While alternative methods exist, this approach remains widely accepted, as it aligns with prior studies and minimizes biases from multi-author collaborations. Potential researcher mobility does not significantly affect its validity, as national funding, institutional resources, and local expertise play a key role. Figure 5 shows the top ten countries of affiliation for the studies we considered. The results for our second research question (RQ2) indicate that the four countries most frequently associated with the studies are the US, with the UK and India tied, followed by Korea and then Australia (c. 19, 31%; 7, 11%; 5, 8%; and 4, 6.5%), respectively. They were followed by authors from Singapore, Japan, and Italy, who contributed approximately 5% each. Russian and Hong Kong researchers came after that, each with a 3.2% share. The remaining articles were published across various countries, each with a frequency of one publication, as illustrated in Figure 5.

**Figure 6:**
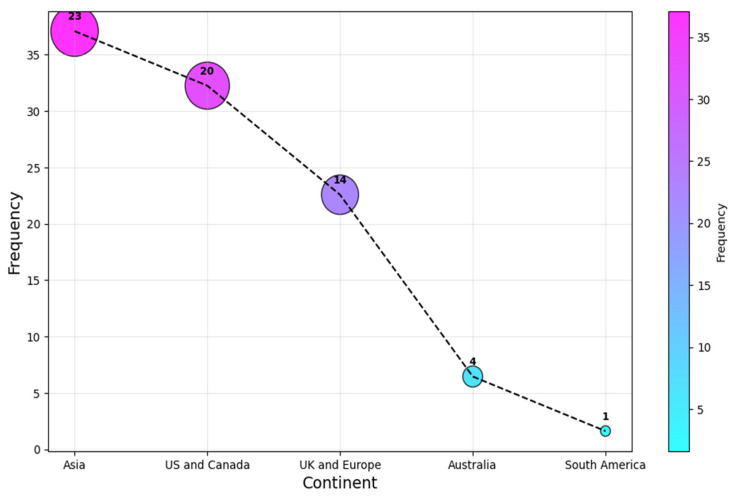
Continent of publication.

In contrast, as a continent, Asia leads the statistics with a (c. 23, 37%) share, followed by the US and Canada, and UK and European researchers contributed (c. 20, 32%) and (c. 14, 23%), respectively. Authors from Australia and South America were the least associated continents for the selected articles, with a share of (c. 4, 6%) and (c. 1, 2%) each. This result is shown in Figure 6. It is clear that the research is relatively concentrated to a selected number of regions, as the four most affiliated countries account for over 60% of all the affiliations. This highlights the need for more research on AI-SaMD across different countries to better understand the impact of sociocultural differences, particularly in Africa, a continent with no published studies in this area.

### 4.3. Analysis Based on Research Strategy and Clinical Environment (RQ3.1 and 3.2)

**Figure 7:**
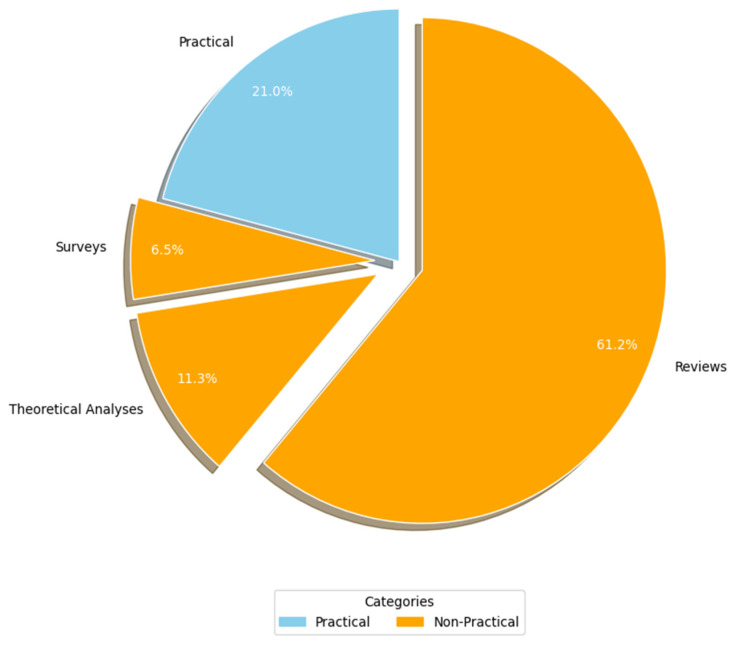
Study strategy used.

Figure 7 (RQ3.1) presents the breakdown of the studies based on the research methodologies employed. Primary studies were categorized into two primary research approaches: practical and non-practical. Practical studies involve implementation or real-world application to generate results (e.g., experiments, case studies, and simulations). In contrast, non-practical studies rely on theoretical analyses, reviews, surveys, and questionnaires to derive insights without direct practical implementation. A significant majority of the AI-SaMD research (c. 49, 79%) adopted non-practical approaches, primarily through reviews (c. 38, 61.3%), theoretical analyses (c. 7, 11.3%), and surveys (c. 4, 6.5%). A potential explanation for the lack of practical studies might come from the fact that working with a real-world case study from healthcare environments is a problem in much research because of confidentiality issues. In other words, publishing details about the participant's healthcare organization, including the specific AI-SaMD outcomes used by that organization, could breach confidentiality. Because most of the articles in this study were non-practical, their data were analyzed qualitatively.

**Figure 8:**
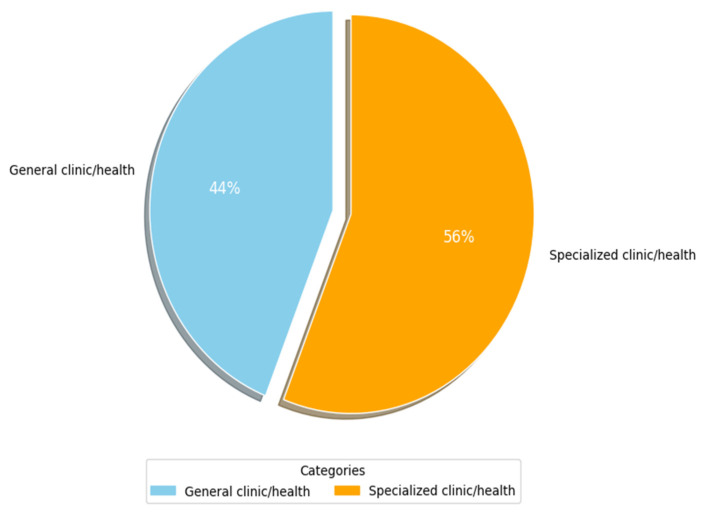
Clinic/medical environment used.

Figure 8 shows the main types of clinics and medical environments that have been used by researchers (RQ3.2). More than 50% of researchers (c. 35, 56%) focused on specialized clinics in their studies. Table 6 lists the specialized clinics that were identified. Columns 3 and 4 show the frequency and percentage of occurrence for each attribute as it appeared in the primary studies, respectively, while column 2 shows the primary study number with sub-categories of such clinics. Herein, the key environments identified are nine. The most widely specialized environment is radiology (c. 11, 17.7%), followed by clinical administration with a share of (c. 5, 8.1%). Next are clinical trials, ophthalmology, and cancer contexts, with a (c. 3, 4.8%) share each. They are followed by dermatology, geriatric and children care, and cardiovascular clinics with a (c. 2, 3.2%) share each. The specialized clinics of kidneys, hypertension, Alzheimer, and nuclear medicine are the least widely considered in the primary studies of this SLR, with a (c., 1, 1.6%) share each. The last column lists examples of clinic/disease categories and their respective study counts. From Table 6, it can be seen that many studies that did not focus on a specialized clinic (i.e., general clinic shown in Figure 8) addressed different issues related to regulatory frameworks and guidelines of AI-SaMD. A plausible explanation for the above findings is that AI-based SaMD has progressed rapidly in radiology diagnostics because image-based data are well-suited for AI applications like pattern recognition, anomaly detection, and diagnostics. AI models thrive in environments where large, structured datasets are available, and medical imaging (CT, MRI, and X-rays) provides this. This makes radiology an ideal testing ground for SaMD solutions. In contrast, areas like kidney diseases, hypertension, and Alzheimer’s often rely on less uniform data, such as lab tests, clinical observations, and patient history, which are harder to standardize and analyze using current AI methods. Additionally, the general health environments have large commercial markets due to their broader applicability. Companies developing AI-based SaMD solutions often prioritize fields with a higher potential return on investment. Fields like Alzheimer’s or kidney diseases, while critical, may not offer the same immediate commercial benefits, slowing down focused research and deployment.

### 4.4. Analysis Based on Challenges (RQ4)

**Figure 9:**
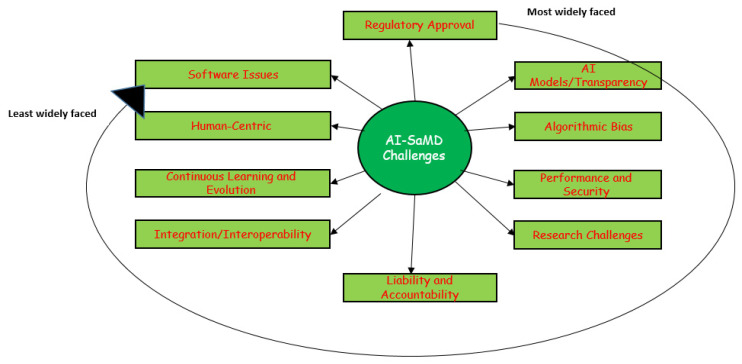
The most widely faced challenges that researchers encountered when using AI-SaMD.

Developing AI-SaMD solutions with retrospective data faces significant challenges. Figure 9 illustrates the top 10 most common issues and difficulties faced by researchers and clinicians when using AI-SaMD (RQ4 in this SLR). They are, in order, regulatory approval, “Black-box” AI models/transparency, algorithmic bias, performance and security, research challenges, liability and accountability, integration/interoperability, continuous learning and evolution, human-centric, and software issues.

Table 7 provides a detailed breakdown of the challenges identified in our study. The first column lists the specific problems, the second column explains each problem in more detail, and the next three columns show the number of the study where the challenge was found, how often it occurred, and the percentage of studies that had this challenge. Due to space constraints, we classified the most significant challenges into four groups.

#### 4.4.1. Regulatory Frameworks Around the World (Challenges 1 and 6)

Approximately 70% of AI-SaMD researchers focus on regulatory issues, as these are crucial not only for technology and health but also for national security (e.g., medical image ownership [75]). AI-SaMDs must comply with local regulations to be marketed and used on patients. In the EU, compliance leads to Conformité Européenne marking, while in the US, the FDA (the Food and Drug Administration (https://www.fda.gov/)) handles approvals. Post-Brexit, the UK introduced the United Kingdom Conformity Assessment mark for medical devices, with a transition period until mid-2023 [76]. Navigating diverse regulatory frameworks is complex for companies, though countries like Switzerland have eased this process by accepting FDA-approved devices [77].

#### 4.4.2. AI Model (Challenges 2 and 3)

AI-SaMDs often rely on black-box solutions, where the underlying algorithms generate outputs without providing clear insights into the decision-making process. This lack of transparency can hinder the assessment of AI-generated results, making it challenging to justify medical decisions, especially in the context of potential malpractice liability claims. To address these concerns, the interpretability and explainability of AI tools have emerged as crucial strategies. Interpretable AI employs transparent or “white-box” algorithms, such as linear models or decision trees, which can be readily understandable. In contrast, explainable AI utilizes a secondary AI algorithm to provide post hoc explanations for the outputs of black-box models. However, current techniques may not be sufficiently robust to explain the complex, individual-level decisions made by black-box AI systems [78,79].

#### 4.4.3. Human-Centric Factors (Challenge 9)

This challenge includes different human and organization factors such as significant inter-expert variability in diagnosing [15], limited clinical understanding of how AI reaches decisions [35], lack of human resources [36], trust between clinicians and AI systems [40], the need for stakeholder collaboration [45], staff training [54], limited user acceptance [60], the need for guidelines on AI-SaMD use [65], and skills related to data governance [36]. Miscommunication is central with these factors. This occurs on two fronts: (1) ineffective communication among stakeholders and (2) miscommunication between healthcare professionals and AI systems. These communication breakdowns contribute to diminished trust in AI-SaMD [40], limited user acceptance [60], and the need for comprehensive staff training [54]. According to [7], misunderstanding often stems from loosely defined terminology. While it is assumed that stakeholders in a meeting are using the same terms with a shared understanding, in reality, their interpretations may vary significantly. This misalignment can lead to prolonged debates, misunderstandings, and poor decision making. Such issues appear to be a facet of human nature and are not exclusive to terminology related to technology [80].

#### 4.4.4. Data Governance (Challenges 4 and 5)

Limited access to large datasets like EHRs is an obstacle in implementing AI-SaMD. This stems from data protection laws such as the GDPR (the General Data Protection Regulation (https://gdpr-info.eu/ (accessed on 30 March 2025)) in Europe and the need for specific patient consent [28]. Public data on dataset validation and testing across algorithms is insufficient, making it impossible to assess generalization or detect potential biases (e.g., demographic bias) [27]. Alternatively, the collection and sharing of extensive datasets via open platforms such as OpenNeuro (OpenNeuro is a free and open platform for sharing neuroimaging data (https://openneuro.org/)) can present significant challenges, notably in terms of ownership and privacy. As shown in Table 6, radiology is the most specialized clinic widely used by researchers. While healthcare facilities in the US generally hold “ownership rights” over imaging data, patients retain certain rights, and their privacy must be protected under laws like HIPAA in the US [81]. Copyright concerns are minimal, as medical images are often not copyrightable, and datasets are typically distributed under CC-0 licenses to avoid legal complications. Ultimately, patient privacy, rather than ownership or copyright, is the primary challenge in sharing medical imaging data [76,81].

### 4.5. Analysis on Researcher Recommendations (RQ5)

There are some recommendations in the current literature that need implementation in real healthcare environments, which can definitely lead to improving AI-SaMD quality. In addition, such recommendations can be a roadmap for further research in this field. Table 8 shows the most important detailed recommendations made by the researchers. Column one lists the recommendations or research directions that were found in our study. Column two briefly provides more details about each recommendation, column three displays the corresponding primary study number, and columns four and five present the frequency and percentage of recommendations.

From Table 8, it is clear that over 50% of the researchers believe the most important recommendation is that related to addressing the regulatory authorities (#1 in Table 8). Regulatory authorities play a role; their involvement ensures that AI-SaMDs are safe, effective, and reliable while fostering innovation and public trust. Currently, their role might be focused on four aspects: (a) establishing clear guidelines and standards, (b) facilitating innovation through flexible pathways, (c) enforcing rigorous post-market surveillance, and (d) ensuring ethical AI use, particularly around patient data privacy, informed consent, and bias reduction. These four tasks are essential to unlocking the potential of AI-SaMD technologies while safeguarding public health and fostering trust. However, the implementation of such tasks is not easy because regulatory authorities, considering that their role is crucial, face challenges such as staying ahead of technological advancements, defining risk-based classifications for AI-SaMD, and balancing innovation with safety. Table 8 also shows that current AI-SaMD researchers recommend the importance of collaborations between diverse stakeholders (e.g., clinicians, IT specialists, regulators, and legal experts). Such an interdisciplinary collaboration is expected to enhance publication dissemination (recommendation 4 in Table 8) through improving research quality, broadening co-authorship networks, and enabling access to shared data, leading to high-impact studies. Collaboration with industry and regulators aligns research with real-world needs, increasing its relevance and visibility. Collaborative researchers also present findings at different conferences, expanding their reach and citation potential. Without a doubt, large-scale, multi-institutional studies attract wider readership and improve dissemination across academic, industry, and policy platforms.

## 5. Limitations and Future Work

Our systematic literature review (SLR) has some limitations. The study only considered papers written in English, excluding research published in other languages. In addition, only peer-reviewed publications were included, while preprints from open-access repositories such as arXiv and PsyArXiv were omitted. Future studies could broaden the scope by incorporating non-English papers, preprint sources, and governmental websites. In addition to the language and database restrictions, it is important to acknowledge a limitation within our search strategy, specifically, the omission of “machine learning” and “deep learning” as keywords. Consequently, some relevant studies may not have been retrieved, particularly those that discuss AI-based methodologies without explicitly mentioning AI. This could have led to the exclusion of certain relevant publications, potentially affecting the comprehensiveness of our review. Future studies may consider refining the search string to capture a broader range of AI-related research. All materials as well as templates used in our review can be provided to other researchers interested in repeating it, but we should note that the changes that occur in digital libraries from time to time might mean that searches are not completely repeatable.

As highlighted in Figure 7, there is a critical need to evaluate the quality of AI-SaMD through practical research methods, such as experimentation, real-world case studies, and simulations; these types of research are under-represented compared to non-practical methods like reviews and theoretical analyses. Increasing the emphasis on practical investigations, as recommended by some researchers (see #4 in Table 8), could significantly enhance the quality and applicability of AI-SaMD publications. A final open point for further research lies in the descriptive nature of the authors’ analyses. Incorporating statistical inference or modeling techniques could enhance the depth of the findings. For instance, developing a predictive model to forecast the annual publication trend would offer a more robust understanding of research output trajectories.

## 6. Conclusions

This study provides a comprehensive summary of state-of-the-art AI-SaMDs based on several attributes. A systematic literature review was conducted, analyzing 62 studies published between 2015 and 2024. Key findings include the following: (1) journals contributed more significantly to this field of research than other publication venues (e.g., conferences, workshops, and symposiums), with the Korean Journal of Radiology emerging as the most common venue; (2) researchers from the United States, the United Kingdom, India, Korea, and Australia were the most active in this area; (3) the predominant research strategy was non-practical, with over 50% of studies focusing on specialized clinical settings, and radiology, clinical administration, trials, ophthalmology, and oncology were the most extensively studied domains; (4) the top 10 challenges of AI-SaMD identified were regulatory approval, lack of transparency in “black-box” AI models, algorithmic bias, performance and security concerns, research challenges, liability and accountability issues, integration and interoperability difficulties, the continuous learning and evolution of AI systems, human-centric factors, and software-related issues; and (5) key recommendations from researchers to address these challenges included fostering interdisciplinary partnerships, providing education and training for clinicians, integrating AI-SaMD with other healthcare systems (e.g., EHRs), and improving the quality of AI-SaMD publications through the enhanced validation of datasets. These findings aim to assist health organizations, AI specialists, compliance officers, and other stakeholders in better understanding the current state and applicability of AI-SaMDs. Additionally, this study offers a solid foundation for researchers and practitioners to address identified challenges in greater detail when developing new AI models. Finally, this article outlines its limitations and suggests promising directions for future research in this rapidly evolving field.

## Figures and Tables

**Table 1:** Differences between SaMD and AI-SaMD.

Aspect	SaMD	AI-SaMD
Technology	Traditional programming	AI algorithms (machine learning and natural processing language)
Adaptability	Static functionality defined at deployment	Dynamic, with potential for continuous learning and improvement
Regulatory	Straightforward regulatory approval process	Requires more (e.g., transparency and bias mitigation).
Validation	Before deployment	Ongoing validation due to learning algorithms
Examples	(a) MRI Image Viewing Application; (b) Computer-Aided Detection (CAD) Software (https://www.fda.gov/medical-devices/software-medical-device-samd/what-are-examples-software-medical-device?utm_source=chatgpt.com (accessed on 30 March 2025))	(a) Arterys Cardio DL to analyze cardiovascular images; (b) EnsoSleep to diagnose sleep disorders (https://medicalfuturist.com/); (c) Apple Watch Sleep Apnea Detection Feature (https://www.investopedia.com/apple-gets-fda-approval-for-smartwatch-sleep-apnea-detection-feature-8713323?utm_source=chatgpt.com (accessed on 30 March 2025))

**Table 2:** RQs with their motivations.

ID	Research Question	Motivation
RQ1	What are the most prominent publication venues for research in AI-SaMD?	To help researchers in finding the main conferences and journals in the AI-SaMD field to publish their research in a suitable outlet
RQ2	Who are the active countries on AI-SaMD?	To highlight the most active countries in AI-SaMD research, providing insights into key contributors and prominent researchers in the field
RQ3	RQ3.1: How do the selected studies conduct their research?	To determine the research design of the study and how the authors analyzed their data
	RQ3.2: In what environments are AI-SaMD studied?	To identify the specific types of clinics and diseases targeted by the studies
RQ4	What are the key challenges in the AI-SaMD’s implementation?	This question addresses the current challenges and explores how they impact the development process
RQ5	What recommendations have researchers provided to the AI-SaMD community for practical application?	This RQ identifies the common recommendations of researchers that could help the AI-SaMD’s community and help researchers in future work

**Table 3:** Distribution of studies following inclusion and exclusion criteria (2015–2024).

Library	Discovered Studies	Inaccessible Studies	Repeated Studies	Exclusive Studies	Primary Studies
IEEE	4	0	2	0	2 studies: [11,12]
PubMed	19	0	1	0	18 studies: [13,14,15,16,17,18,19,20,21,22,23,24,25,26,27,28,29,30]
Semantic Scholar	41	2	14	9	16 studies: [31,32,33,34,35,36,37,38,39,40,41,42,43,44,45,46]
Springer	25	2	6	1	16 studies: [47,48,49,50,51,52,53,54,55,56,57,58,59,60,61,62]
WoS	39	2	25	2	10 studies: [63,64,65,66,67,68,69,70,71,72]
Total	128	6	48	12	62

**Table 4:** Study distribution by publication venue.

Library	Journal	Conference	Workshop	Book Chapter	Total
IEEE	0	2	0	0	2
PubMed	18	0	0	0	18
Semantic Scholar	13	1	1	1	16
Springer	16	0	0	0	16
WoS	10	0	0	0	10
Total	57	3	1	1	62

**Table 5:** Publication venues with multiple selected studies.

Venue	Freq.
Korean Journal Radiology	3
npj Digital Medicine	2
International Journal of Computer Assisted Radiology and Surgery	2
Healthcare—MDPI	2
Australian Journal of Dermatology	2
Emergency Radiology	2

**Table 6:** The primary environments in which the researchers applied AI-SaMD.

#	Major Clinic	Subspecialty	Freq.	%	Study Number and Sub-Subspecialty
1	General	N/A	26	42	[12,13,17,19,20,21,22,23,24,28,30,32,34,36,38,39,40,41,44,52,55,56,57,62,64,67,71] Regulatory frameworks [12,13,19,20,21], general surgery [17], public health [36], and effect of AI in healthcare [54]
2	Subspecialty	Radiology	11	17.7	[16,18,24,27,31,42,58,59,60,63,70] Neuroradiology [15], chest X-ray [18], MRI [30], and trauma radiology [60]
3		Clinical administration	5	8.1	[35,43,45,48,66] Decision making [35,43,45,48,66] and workflow [48]
4		Clinical trials	3	4.8	[11,50,54]
5		Ophthalmology	3	4.8	[14,15,46]
6		Cancer	3	4.8	[29,33,72]
7		Dermatology	2	3.2	[37,68]
8		Geriatric and children care	2	3.2	[49,61]
9		Cardiovascular	2	3.2	[25,69]

**Table 7:** The common challenges of AI-SaMD.

#	Challenge	Description	Study Number	Freq.	%
1	Regulatory Approval	AI-SaMD must meet stringent regulatory requirements (e.g., FDA and EMA) for safety and efficacy, often challenged by the dynamic and adaptive nature of AI models.	[11,12,13,16,19,20,22,25,26,27,28,29,30,32,34,35,36,37,38,40,41,43,44,45,50,52,54,57,61,62,63,64,67,71]	34	54.8
2	“Black-box” AI Models/Transparency	Complex AI algorithms, especially deep learning, lack interpretability, making it difficult to explain how decisions are made, which regulators and clinicians demand.	[16,17,18,24,27,30,31,35,50,51,54,55,56,57,58,59,60,61,68,70,72]	21	33.9
3	Algorithmic Bias	Training data biases can lead to AI models performing unequally across demographic groups, risking unfair or unsafe outcomes.	[21,24,26,27,30,35,37,38,40,45,46,55,56,59,61,65,66,67,68,69]	20	32.3
4	Performance and Security	AI-SaMD must reliably operate under varying conditions and safeguard against cybersecurity threats that could compromise patient safety.	[13,16,21,22,28,30,41,44,45,46,48,56,57,62,65,69,71]	17	27.4
5	Research Challenges	Limited access to high-quality, diverse, and sufficiently large datasets can hamper model training and validation, impacting performance and generalizability.	[11,18,27,28,29,31,37,40,41,42,45,50,58,60,67,69]	16	25.8
6	Liability and Accountability	Ambiguity around who is responsible for errors (developer, deployer, or clinician) complicates legal and ethical accountability in AI-SaMD.	[14,16,18,27,30,34,51,65,67]	9	14.5
7	Integration/Interoperability	AI-SaMD must seamlessly integrate with existing clinical workflows, EHRs, and other systems for practical deployment.	[28,30,47,48,52,53,57,63,70]	9	14.5
8	Continuous Learning and Evolution	AI models that update with new data post-deployment require rigorous monitoring to ensure consistent compliance with safety and regulatory standards.	[13,21,24,26,39,43,68,69,70]	9	14.5
9	Human-Centric	Critical human/organizational factors can impede the effective integration of AI-SaMD into practice. They emphasize the need for strategies addressing trust, understanding, and collaboration to bridge the gap between technology and real-world application.	[15,35,36,40,45,54,60,63]	8	12.9
10	Software Business	This includes software updating, vendor support, system documentation, licensing, and software upgrading [74]. Such activities must be carefully managed to maintain compliance, functionality, and compatibility while avoiding system disruptions.	[19,46,52,62]	4	6.5

**Table 8:** The suggestion most commonly recommended by researchers for implementation in clinical settings.

#	Recommendation	Description	Study Number	Freq.	%
1	Addressing regulatory challenges in AI-SaMD	As mentioned in answering RQ4, developed countries like the US and Europe have already adopted regulatory frameworks. Additional frameworks exist elsewhere, such as the PMDA (Pharmaceuticals and Medical Devices Agency (https://www.pmda.go.jp/english/ (accessed on 30 March 2025)) in Japan. In the near future, we should focus on the less-developed countries, especially those not depicted in Figure 4 and Figure 5, that face a crucial decision. Should they adopt an existing regulatory framework or develop their own? Each option carries distinct national implications.	[12,15,18,19,20,21,22,23,26,27,29,30,36,37,38,39,40,41,43,44,45,52,54,55,57,61,62,64,66,67,69,71]	32	51.6
2	AI algorithms	Medical malpractice liability associated with the use of AI in clinical practice remains unresolved, with legal frameworks still in flux. Furthermore, interpretable AI is preferred when it achieves performance comparable to black-box models. If not, black-box models might be used, but only after extensive testing and clinical trials. This research direction aims at conducting a comprehensive comparison between AI-SaMD black-box and white-box algorithms across various quality attributes. Additionally, it is crucial to establish minimum requirements for the use of black-box algorithms in scenarios where they are the only viable option.	[24,30,31,32,34,35,45,46,48,53,59,61,62,65,68,69,72]	17	27.4
3	Interdisciplinary partnership and training	To enhance the adoption of AI-SaMD, fostering stronger partnerships between academia and industry is crucial. Equally important is equipping clinicians with targeted education and training on the principles, applications, and limitations of AI-SaMD, ensuring they can confidently integrate these tools into clinical practice and academic programs by institution. Increased collaboration among diverse stakeholders—including clinicians, IT specialists, regulators, and legal experts—can further accelerate progress. By sharing AI-SaMD methods, skills, experiences, and data, this collaborative approach can foster comparative research among national and international health institutes.	[16,17,36,41,45,49,50,55,57,63,66,69]	12	19.4
4	Other recommendations	Integration with the other enterprise systems	[33,41,51,54,60]	5	8.1
		More validation	[25,28,42,47]	4	6.5
		Enhancing AI-SaMD publication	[58,60,70]	3	4.8

## Data Availability

Data are contained within the article.

## Appendix A. Data Extraction Form

**Section 1: Paper information**	
Author	Author(s) of the study
Name	Title of the study
Type	Publication types: journal, conference, workshop, or symposium
Country	The place of the study
Year	The year of the study
**Section 2: Quality assessment**	
The rationale for the study in the introduction clear?	Yes/No
The design/methodology of the study appropriate?	Yes/No
The findings and results of study are clearly stated?	Yes/No
The findings of the study are evaluated properly?	Yes/No
**Section 3: Data extraction**	
Methodology	It can be either practical or non-practical. If the study relies on observation, it is practical; otherwise, it is non-practical. Experiments, simulations, and real-world case studies are examples of practical studies, while reviews, theoretical analyses, surveys, and questionnaires are examples of non-practical studies.
Objective	Objectives/aims of the study.
Challenges in implementation of AI-SaMD	List the difficulties that researchers and clinicians often face when using AI-SaMD.
Quality attribute	The AI-SaMD quality attributes that were supposed to be measured and validated (e.g., safety).
Measure	The metrics and validation approaches used to measure considered quality attributes.
Context	It includes the specific types of clinics and diseases targeted by the studies.
Data analysis	Whether quantitative or qualitative.
Results	The subjective results of the study.
